# Machine Learning Classification of Current Functional Impairment in Older Adults with Diabetes: Evidence from CHARLS 2015

**DOI:** 10.3390/jcm15156062

**Published:** 2026-08-04

**Authors:** Qi-Shuai Ma, Li-Qun Jiang, Buong-O Chun

**Affiliations:** Graduate School of Physical Education, Myongji University, Yongin 17058, Republic of Korea; maqishuai@mju.ac.kr (Q.-S.M.); jiangliqun@mju.ac.kr (L.-Q.J.)

**Keywords:** machine learning, cross-sectional classification, functional impairment, diabetes, older adults, CHARLS, SHAP

## Abstract

**Background**: Functional impairment is common among older adults with diabetes, but multidimensional machine learning classification in this population remains underexplored. This study compared machine learning algorithms for identifying concurrent functional impairment among older adults with self-reported physician-diagnosed diabetes. **Methods**: This cross-sectional study included 1213 adults aged ≥ 65 years from the nationally sampled 2015 China Health and Retirement Longitudinal Study cohort. Functional impairment was defined as limitations in ≥2 activities of daily living or instrumental activities of daily living. Training set feature selection yielded 18 input features. Eight algorithms were evaluated in a held-out internal test set. Repeated stratified nested 10-fold cross-validation with five repeats assessed stability conditional on the locked feature set. SHAP assessed feature contributions and ranking stability. **Results**: Functional impairment was present in 34.21% of participants. Random forest achieved the numerically highest test-set AUC (0.774; 95% CI, 0.714–0.833), with sensitivity of 0.512 and a Brier score of 0.181. Its advantage over other models was modest. Under repeated nested cross-validation, random forest achieved a mean AUC of 0.808 ± 0.043 and a mean Brier score of 0.166 ± 0.016. SHAP rankings were stable (Kendall’s W = 0.901); leading contributors included depressive symptoms, self-rated health, 2.5-m walking test completion time, distance vision, history of falls, executive function, and bilateral grip strength. Excluding direct physical performance inputs retained most discrimination but modestly reduced threshold-dependent performance. **Conclusions**: Random forest provided the most favorable overall internal performance for classifying concurrent functional impairment, although sensitivity remained modest. External and prospective validation is required before clinical implementation.

## 1. Introduction

Diabetes has become one of the most important chronic disease burdens worldwide. According to the 10th edition of the International Diabetes Federation (IDF) Diabetes Atlas, approximately 537 million adults aged 20–79 years were living with diabetes globally in 2021, corresponding to a prevalence of 10.5%, and this number is projected to rise to 783 million by 2045. Notably, the prevalence among individuals aged 75–79 years had already reached 24.0%, indicating that the burden of diabetes is becoming increasingly concentrated in older populations [1]. In China, nationally representative studies, including the China Health and Retirement Longitudinal Study (CHARLS), have likewise shown a continuing increase in diabetes prevalence and related health risks among middle-aged and older adults, suggesting that future demands for functional support and caregiving may further expand and place sustained pressure on healthcare and long-term care systems [2,3]. Among older adults with diabetes, functional impairment is typically manifested as limitations in activities of daily living (ADL) and instrumental activities of daily living (IADL), and represents one of the most clinically meaningful health outcomes. Previous systematic reviews and meta-analyses have shown that diabetes is significantly associated with an increased risk of physical disability and functional limitations [4]. Several prospective studies have further indicated that diabetes may accelerate the progression of ADL/IADL limitations and is closely associated with increased risks of falls, hospitalization, long-term care needs, and mortality [5,6]. Therefore, early identification of functional impairment in older adults with diabetes is of substantial importance for promoting healthy aging and optimizing clinical management.

The development of functional impairment in older adults with diabetes is clearly multidimensional and cannot be adequately explained by any single indicator. At the physical level, diabetes-related chronic inflammation, microvascular dysfunction, and metabolic abnormalities may accelerate declines in skeletal muscle mass and strength, thereby leading to reduced grip strength and slower gait speed; these processes also represent key shared pathways underlying sarcopenia, frailty, and functional disability [7,8]. At the sensory and cognitive levels, diabetes is associated with an increased risk of cognitive impairment and dementia, while declines in sensory function, such as vision and hearing, may further amplify the risk of ADL/IADL limitations [9,10]. At the psychological level, diabetes and depression have a bidirectional relationship, and older adults with diabetes comorbid with depression often face a higher risk of functional disability [11,12]. A longitudinal study based on CHARLS further suggested that depressive symptoms and ADL impairment may also be bidirectionally related [13]. In addition, falls and related factors should not be overlooked. Previous studies have shown that older adults with diabetes are at greater risk of falls than their non-diabetic counterparts, with hypoglycemia, retinopathy, peripheral neuropathy, and sensory–balance impairment all potentially contributing to this increased risk; a history of falls is itself an important warning sign for subsequent functional decline [14,15]. Overall, functional impairment in older adults with diabetes is more likely to result from the combined effects of multidimensional risk factors spanning physical, sensory, cognitive, psychological, and safety-related domains.

Traditional multivariable logistic regression has been widely used in studies of functional outcomes in older adults, but it has certain limitations in handling large numbers of candidate variables, potential nonlinear relationships, and complex interactions. In recent years, machine learning (ML) methods have increasingly been applied to the identification and modeling of functional status in older populations. For example, several studies based on CHARLS have used algorithms such as random forest, XGBoost, and multilayer perceptron to identify disability or ADL limitation, and have incorporated Shapley Additive Explanations (SHAP) to interpret model performance, showing that variables such as self-rated health, depressive symptoms, sleep duration, grip strength, and respiratory function contribute substantially to prediction [16,17,18,19]. However, existing research has still primarily focused on the general older population or specific comorbidity subgroups, whereas studies specifically developing models to identify functional impairment in older adults with diabetes remain relatively limited. At the same time, considerable heterogeneity persists across studies in feature selection procedures, approaches to class imbalance, control of data leakage, the rigor of internal validation, and the assessment of model interpretability and stability [16,19]. These gaps highlight the need for a more rigorous methodological framework to systematically investigate the classification of functional impairment in the more clinically targeted population of older adults with diabetes.

Accordingly, the present study aimed to develop and compare machine learning models for the cross-sectional classification of current functional impairment among older adults with self-reported diabetes using CHARLS 2015 data. The objective was to identify features associated with the model output at the same survey wave, rather than to predict future functional decline or establish causal determinants. We also evaluated calibration, decision-curve net benefit, internal validation performance, and the stability of SHAP-based feature contributions.

## 2. Methods

### 2.1. Data Source and Study Population

**Figure 1:**
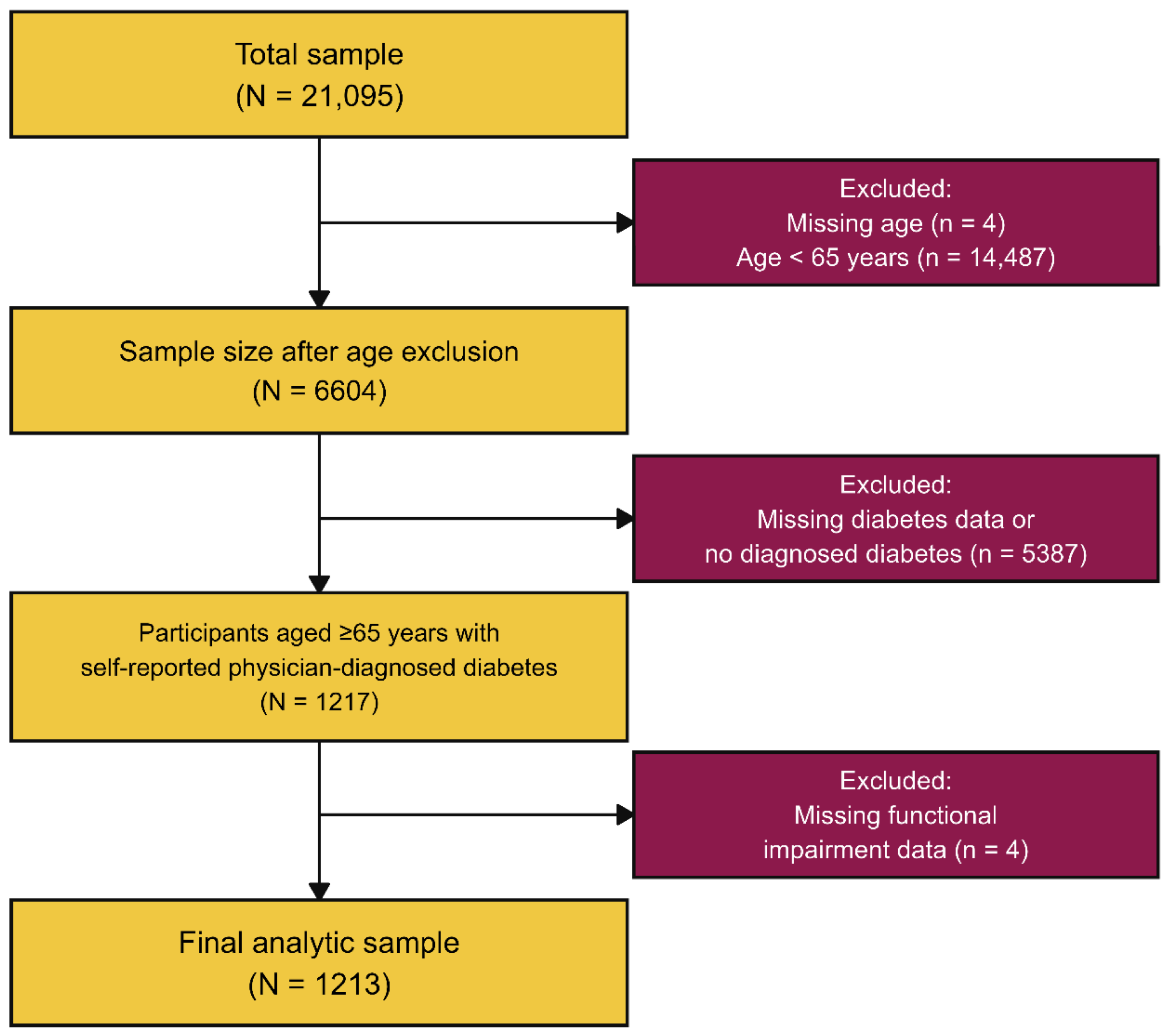
Flowchart of participant selection.

Data were derived from the 2015 wave of the China Health and Retirement Longitudinal Study (CHARLS), which included 21,095 participants. Participants with missing age information or an age below 65 years were excluded, leaving 6604 participants aged ≥ 65 years. Among these participants, those with missing diabetes information or without self-reported physician-diagnosed diabetes were excluded, leaving 1217 eligible participants. Four participants with missing functional-impairment outcome data were subsequently excluded, resulting in a final analytic sample of 1213 participants aged ≥ 65 years with self-reported physician-diagnosed diabetes. This final analytic sample represented an unweighted subgroup of the nationally sampled CHARLS cohort, and CHARLS sampling weights were not incorporated into the machine learning models. The final analytic sample was randomly divided using outcome-stratified sampling into a training set of 966 participants (79.64%) and a held-out internal test set of 247 participants (20.36%). The participant-selection and data-splitting procedures are shown in Figure 1.

### 2.2. Measurement and Definition of Candidate Variables

Candidate variables were obtained from the 2015 CHARLS modules covering demographic characteristics, health status and disease history, anthropometric and laboratory measurements, lifestyle and physical activity, sensory and cognitive function, psychological status, oral health, and physical performance. The candidate variables included sleep, self-reported physical activity, sensory function, cognitive performance, psychological symptoms, grip strength, pulmonary function, walking test performance, comorbidities, and metabolic biomarkers. Variables were analyzed as binary, ordinal, or continuous measures according to their original measurement scales.

Binary variables were coded as 0 or 1, whereas ordinal variables retained the original CHARLS category structure and value-label direction. Self-rated health was coded from 1 (very poor) to 5 (very good). Distance vision, near vision, and hearing were coded from 1 (poor) to 5 (excellent), with higher values indicating better sensory function. No additional reverse coding was performed.

Continuous variables were retained in their original measurement units. Age was measured in years; height and body weight were measured in meters and kilograms, respectively; body mass index (BMI) was expressed in kg/m^2^; waist circumference was expressed in centimeters; and glucose, triglycerides, and HDL cholesterol were expressed in mg/dL. The 2.5-m walking test completion time was calculated as the mean completion time of the two walking trials and expressed in seconds. Higher values indicated longer completion times and poorer walking performance.

BMI was calculated as body weight in kilograms divided by height in meters squared. The triglyceride–glucose (TyG) index was calculated as ln[(triglycerides [mg/dL] × glucose [mg/dL])/2], and the TyG-BMI was calculated as the product of the TyG index and BMI.

Data-quality checks were conducted before feature selection and model development. One observation with an unverifiable height of 0.485 m was identified. For this observation, height and the corresponding BMI and TyG-BMI values were treated as missing. Three additional observations had completion times of 0.02 s in both walking trials; the two trial measurements and their calculated mean completion time were therefore treated as missing. The recorded body weight was retained because an independent source-record error could not be confirmed. After quality control, the observed BMI ranged from 13.86 to 47.01 kg/m^2^, with a median of 24.40 (IQR, 21.97–26.80), whereas TyG-BMI ranged from 118.51 to 452.17, with a median of 222.96 (IQR, 195.52–249.61).

Other anthropometric and laboratory measurements were retained in their original form unless a source-specific coding or measurement-unit error was confirmed. No arbitrary winsorization or distribution-based exclusion was applied. Remaining missing predictor values were handled using the training set-based imputation procedure described below.

### 2.3. Definition of Diabetes

Diabetes status was defined on the basis of self-reported physician diagnosis from the chronic disease module of CHARLS 2015 (“Doctor Told Disease”). Participants who reported having previously been told by a doctor that they had diabetes were classified as having diabetes (coded as 1); all others were classified as non-diabetic (coded as 0). This definition reflects self-reported prior diagnosis and does not constitute a renewed diagnosis based on laboratory indicators in the present study.

### 2.4. Definition of Functional Impairment

Functional impairment was operationally defined using ADL and IADL items from the health and functioning module of CHARLS 2015. For each item, participants reporting difficulty, requiring assistance, or being unable to perform the task were classified as impaired on that item. The total number of impaired ADL/IADL items was then calculated after binarization. Consistent with previous studies [20,21], a total of 0–1 impaired items was defined as no functional impairment, whereas ≥2 impaired items was defined as functional impairment. This indicator reflects the level of functional limitation across the ADL/IADL domains rather than a clinical diagnosis of a single disease entity.

This definition represents the level of functional limitation measured at the same survey wave and was used as a cross-sectional binary classification outcome; it was not treated as a prospective endpoint.

### 2.5. Data Preprocessing and Missing Data Handling

This analysis used cross-sectional data from the 2015 wave of CHARLS. All candidate input variables and the outcome, ADL/IADL impairment, were assessed during the same interview.

After excluding participants with missing outcome data, the remaining 1213 participants were randomly divided into a training set (*n* = 966; 79.64%) and a held-out internal test set (*n* = 247; 20.36%) in an approximately 8:2 ratio, using stratification on the outcome. The held-out test set was sampled from the same CHARLS 2015 source population and was not an externally sourced validation cohort.

Missingness screening was conducted using the training set only. Variables with more than 40% missingness in the training set were excluded before imputation and downstream feature selection procedures. These variables comprised measures of vigorous, moderate, and light physical activity, regular exercise, and total physical activity volume. Consequently, these activity-related measures were not entered into the subsequent consensus ranking, VIF screening, or recursive feature elimination procedures. For descriptive analyses, variable-specific effective denominators were retained and reported when missing observations were present [22,23,24].

To minimize data leakage, all imputation rules were developed using the training set only and then applied unchanged to the held-out test set. Continuous variables were imputed using the training set median, whereas binary and ordinal categorical variables were imputed using the training set mode, with the original 0/1 coding and ordinal category structure retained. Missingness indicators were additionally created for glucose, glycated hemoglobin, creatinine, and C-reactive protein, with a value of 1 indicating a missing original measurement and 0 indicating an observed measurement. These indicators were included in the initial candidate-feature pool but were not retained in the final 18-predictor set.

### 2.6. Statistical Analysis

Baseline characteristics were summarized using descriptive statistics. Continuous variables are presented as mean ± standard deviation (SD), and categorical variables as number (percentage). Participants were classified into non-impaired and impaired groups according to functional impairment status. Between-group differences in continuous variables were assessed using Welch’s *t*-test, whereas categorical variables were compared using Pearson’s χ^2^ test or Fisher’s exact test when appropriate. All tests were two-sided, and *p* < 0.05 was considered statistically significant.

Feature selection was performed exclusively in the development training set. The 50 candidate features were ranked using three complementary approaches: multivariable logistic regression based on the absolute z value, LASSO logistic regression based on the absolute coefficient at the lambda.1se criterion, and random forest based on permutation importance. The mean rank across the three approaches was used as the integrated ranking criterion, and the 30 highest-ranked features were carried forward.

Spearman correlations among the 30 highest-ranked features were examined using an absolute pairwise correlation threshold of 0.90. Multicollinearity was then assessed using variance inflation factors (VIFs), with features exceeding a VIF of 5 removed recursively. Global cognitive function and TyG-BMI were removed during VIF screening. Random forest recursive feature elimination with 5-fold cross-validation and accuracy as the optimization criterion was subsequently used to determine the final feature set size.

### 2.7. Model Development

Eight machine learning classification algorithms were developed using the same locked 18-feature set: logistic regression, LASSO logistic regression, elastic net, random forest, extreme gradient boosting (XGBoost), generalized additive model (GAM), decision tree, and radial support vector machine (SVM). All models were trained using the observed class distribution without class-balancing resampling. This choice was made because class-imbalance corrections may distort probability estimates and may adversely affect the performance of prediction models [25,26].

Logistic regression, LASSO logistic regression, elastic net, GAM, and SVM used standardized inputs; the centering and scaling parameters were estimated from the development training set and applied unchanged to the held-out internal test set. Random forest, XGBoost, and decision tree used inputs on their post-imputation original scales. LASSO tuning used 10-fold cross-validation to select lambda.min. Elastic-net tuning evaluated alpha values of 0.1, 0.3, 0.5, 0.7, and 0.9, with lambda selected by 10-fold cross-validation. Random forest tuning evaluated mtry values of 3, 5, 7, and 9 using 5-fold cross-validation with AUC as the selection metric and 500 trees. XGBoost tuning evaluated prespecified combinations of tree depth, learning rate, and boosting rounds using 5-fold stratified cross-validation. The GAM used restricted maximum likelihood estimation, the decision tree was pruned using the complexity parameter associated with minimum cross-validated error, and radial SVM cost and gamma were selected by 5-fold cross-validation.

Each fitted model generated a probability of concurrent functional impairment. A prespecified probability threshold of 0.50 was used to derive binary classifications for threshold-dependent performance metrics.

### 2.8. Model Evaluation and Interpretation

The primary model comparison was performed once in the held-out internal test set. Discrimination and threshold-based performance were summarized using the area under the receiver operating characteristic curve (AUC), accuracy, sensitivity, specificity, precision, and F1 score. AUCs were reported with DeLong 95% confidence intervals where applicable. Probabilistic error was assessed using the Brier score, calibration was examined using calibration curves, and decision curve analysis was used to describe potential net benefit across probability thresholds [27,28,29]. This evaluation concerned classification of concurrent functional impairment and did not constitute external validation or prospective prediction.

As a secondary stability analysis, all eight algorithms were evaluated using five repeats of stratified nested 10-fold cross-validation across the full analytic sample, yielding 50 outer test folds. This analysis was conditional on the completed dataset and the 18-feature set selected in the original development training set. Feature selection and missing-data imputation were therefore not repeated within the outer folds. Within each outer split, standardization parameters and model-specific hyperparameters were estimated using the outer training data only and then applied to the corresponding outer test fold. The original class distribution and the 0.50 classification threshold were retained. Performance was summarized as mean ± SD across the 50 outer test folds. These results were interpreted as internal resampling stability conditional on the locked model specification, rather than as external validation or a replacement for the primary held-out test-set evaluation.

A prespecified sensitivity analysis evaluated the random forest model after excluding the three direct physical performance inputs: 2.5-m walking test completion time and left- and right-hand grip strength. The reduced-input model was evaluated in the held-out internal test set and under the same repeated nested cross-validation design to assess how much classification performance could be retained when these measurements were unavailable.

After completion of performance evaluation, the selected random forest algorithm was refitted on the full analytic sample solely for interpretation. The 18-feature set and development-selected hyperparameters were locked (mtry = 3; ntree = 500), and no additional full-sample tuning was performed. SHAP values were estimated using 50 Monte Carlo permutations and a stratified background sample of 200 participants. Global importance was summarized using mean absolute SHAP values, feature-contribution direction was displayed using a beeswarm plot, and representative local contributions were illustrated using a waterfall plot [19]. No performance estimate was derived from this full-sample interpretive refit.

SHAP-ranking stability was additionally assessed using the same 50 outer folds as the repeated nested cross-validation. In each fold, the random forest was fitted to the outer training data using the fold-specific mtry selected by inner cross-validation, and SHAP values were calculated only for the corresponding outer test fold. Ranking agreement was summarized using Kendall’s coefficient of concordance, pairwise Spearman correlations, correlations with the full-sample ranking, and top-ranking frequencies. SHAP values were interpreted as contributions to the model-predicted probability of concurrent functional impairment, not as causal effects, independent risk factors, or estimates of future risk.

## 3. Results

### 3.1. Baseline Characteristics

Table 1 summarizes the baseline characteristics of the 1213 participants, including 798 in the non-impaired group and 415 in the impaired group. Compared with participants without functional impairment, those with functional impairment were older and were more likely to be female and to have a higher burden of comorbid conditions, including chronic disease, heart disease, stroke, arthritis or rheumatism, kidney disease, psychiatric or emotional problems, memory-related disease, lung disease, digestive disease, and asthma. They also had a higher prevalence of falls and showed less favorable profiles in several behavioral and functional indicators. Specifically, the impaired group had a longer 2.5-m walking test completion time than the non-impaired group (4.82 ± 2.91 vs. 3.58 ± 1.31 s, *p* < 0.001), indicating slower walking performance among participants with functional impairment. In addition, the impaired group had shorter sleep duration, higher depressive symptom scores, lower memory, executive function, and global cognitive function scores, lower bilateral handgrip strength, and lower peak expiratory flow. By contrast, several metabolic and anthropometric indicators, including blood pressure, body mass index, waist circumference, glucose, glycated hemoglobin, triglyceride-glucose index, triglycerides, HDL cholesterol, LDL cholesterol, and hemoglobin, did not differ significantly between the groups. These findings suggest that functional impairment was associated with broader disadvantages across demographic, clinical, behavioral, and functional domains at baseline.

### 3.2. Candidate Variable Screening and Feature Optimization

The analytic sample was divided into a training set (*n* = 966; 637 non-impaired and 329 impaired) and a held-out internal test set (*n* = 247; 161 non-impaired and 86 impaired). Candidate predictor screening and feature selection were conducted exclusively within the training set. Before feature ranking, five physical activity-related variables—vigorous physical activity, moderate physical activity, light physical activity, regular exercise, and the total physical activity metabolic equivalent of task (MET) score—were excluded because each had more than 40% missing data in the training set. Three additional low-information variables—any chronic disease, psychiatric or emotional problems, and cancer or malignant tumor—were excluded through near-zero-variance screening. Four missingness indicators corresponding to glucose, glycated hemoglobin, creatinine, and C-reactive protein were retained as candidate predictors, resulting in a total of 50 candidate predictors for feature ranking. Missing values in the remaining predictors were imputed using parameters estimated exclusively from the training set. The same imputation parameters were subsequently applied without modification to the held-out internal test set to prevent information leakage.

**Figure 2:**
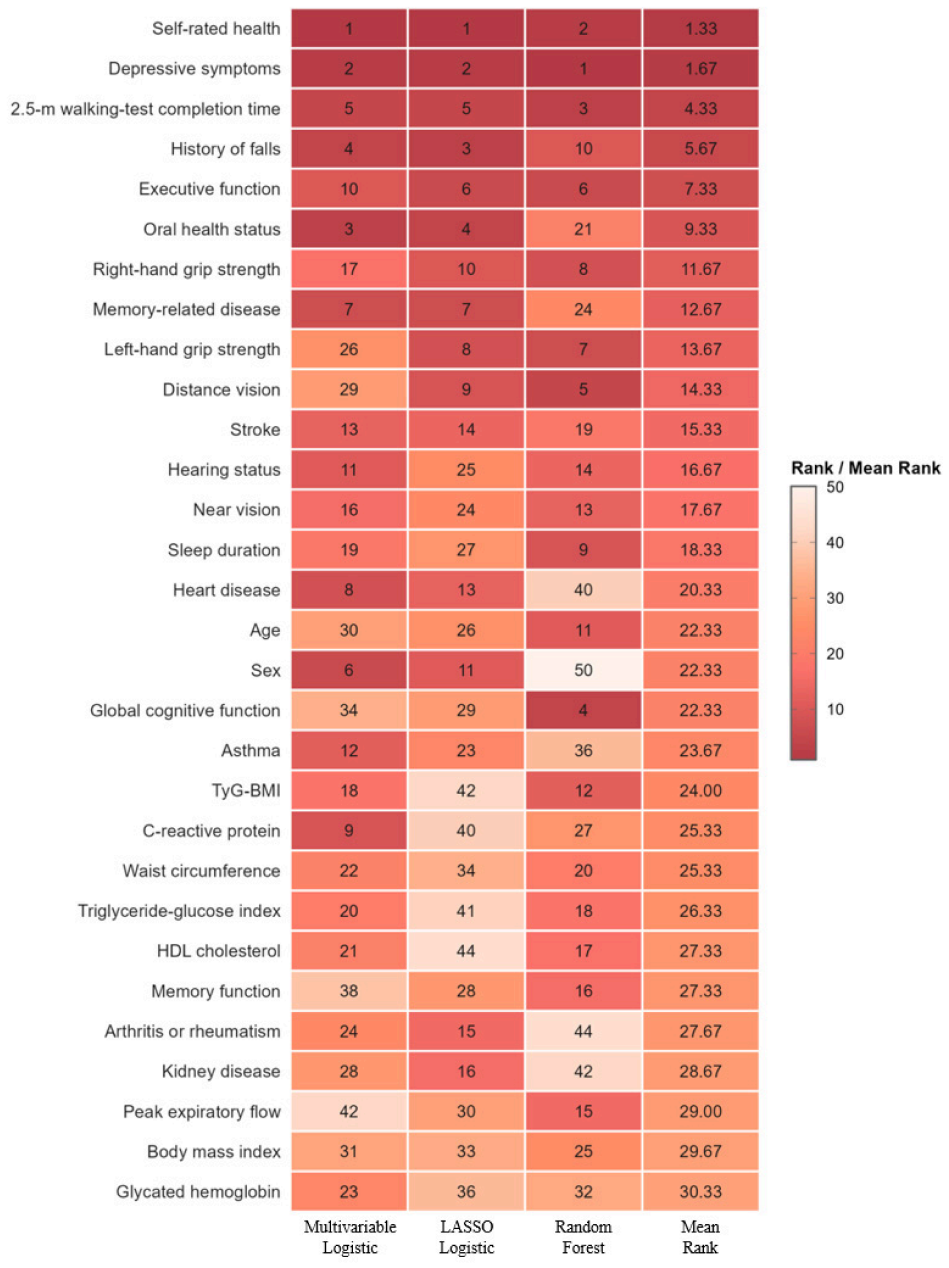
Integrated ranking heatmap of the top 30 candidate variables.

Figure 2 presents the integrated ranking of the 30 highest-ranked candidate predictors obtained from multivariable logistic regression, LASSO logistic regression, and random forest. Lower numerical ranks indicate greater importance within each method, and the mean rank represents the average rank across the three methods. Self-rated health and depressive symptoms ranked first and second overall, respectively, followed by 2.5-m walking test completion time, history of falls, executive function, and oral health status. Right-hand grip strength, memory-related disease, left-hand grip strength, and distance vision were also among the ten highest-ranked predictors. Some predictors showed method-specific variation in their rankings. For example, global cognitive function ranked fourth in the random forest analysis but substantially lower in the multivariable logistic regression and LASSO logistic regression analyses. The 30 highest-ranked predictors were subsequently carried forward for correlation analysis, multicollinearity assessment, and further feature reduction.

**Figure 3:**
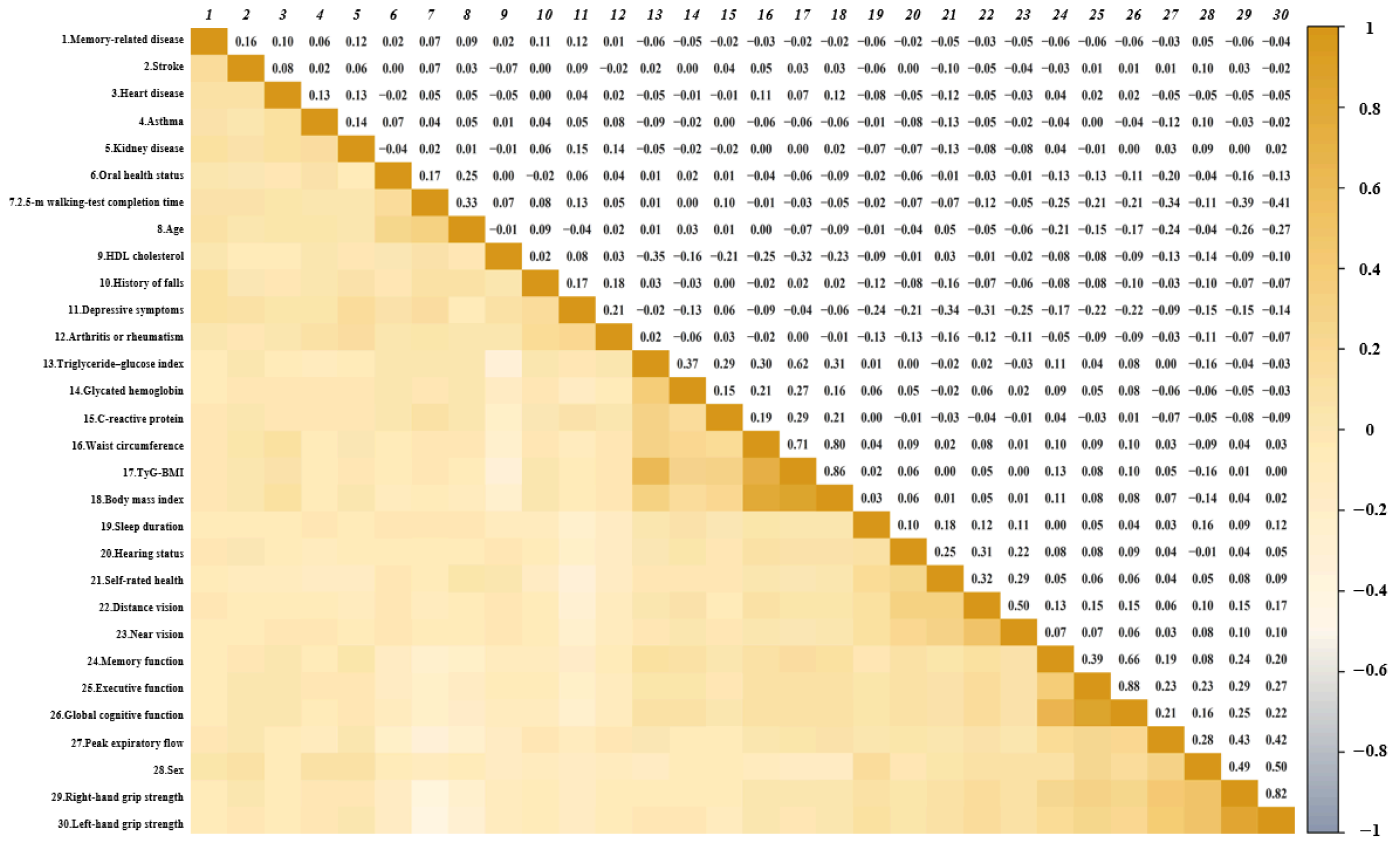
Spearman correlation matrix of the 30 highest-ranked candidate predictors after training set-based screening.

Among these 30 predictors, Spearman correlation analysis showed that the strongest absolute pairwise correlation was between executive function and global cognitive function (|ρ| = 0.877). No absolute pairwise Spearman correlation coefficient exceeded 0.90 (Figure 3); therefore, no predictor was excluded solely on the basis of pairwise correlation. Multicollinearity was subsequently assessed using variance inflation factors (VIFs), with predictors exceeding a VIF threshold of 5 removed recursively.

Subsequent multicollinearity screening and recursive feature elimination further reduced the 30 highest-ranked candidate predictors. During the recursive VIF procedure, global cognitive function and the triglyceride–glucose body mass index were sequentially removed because their VIF values exceeded the prespecified threshold of 5, leaving 28 predictors for recursive feature elimination. Random forest-based recursive feature elimination was then performed in the training set using five-fold cross-validation. Candidate subsets containing 5, 6, 7, 8, 9, 10, 12, 15, 18, 20, and 28 predictors were evaluated, with mean classification accuracy specified as the optimization criterion. Among the evaluated subset sizes, the 18-predictor subset achieved the highest cross-validated accuracy (0.7649) and Cohen’s kappa value (0.4423). Although the performance of the 20-predictor subset was nearly identical, the 18-predictor subset achieved a marginally higher accuracy with fewer input variables and was therefore selected as the final feature set.

**Figure 4:**
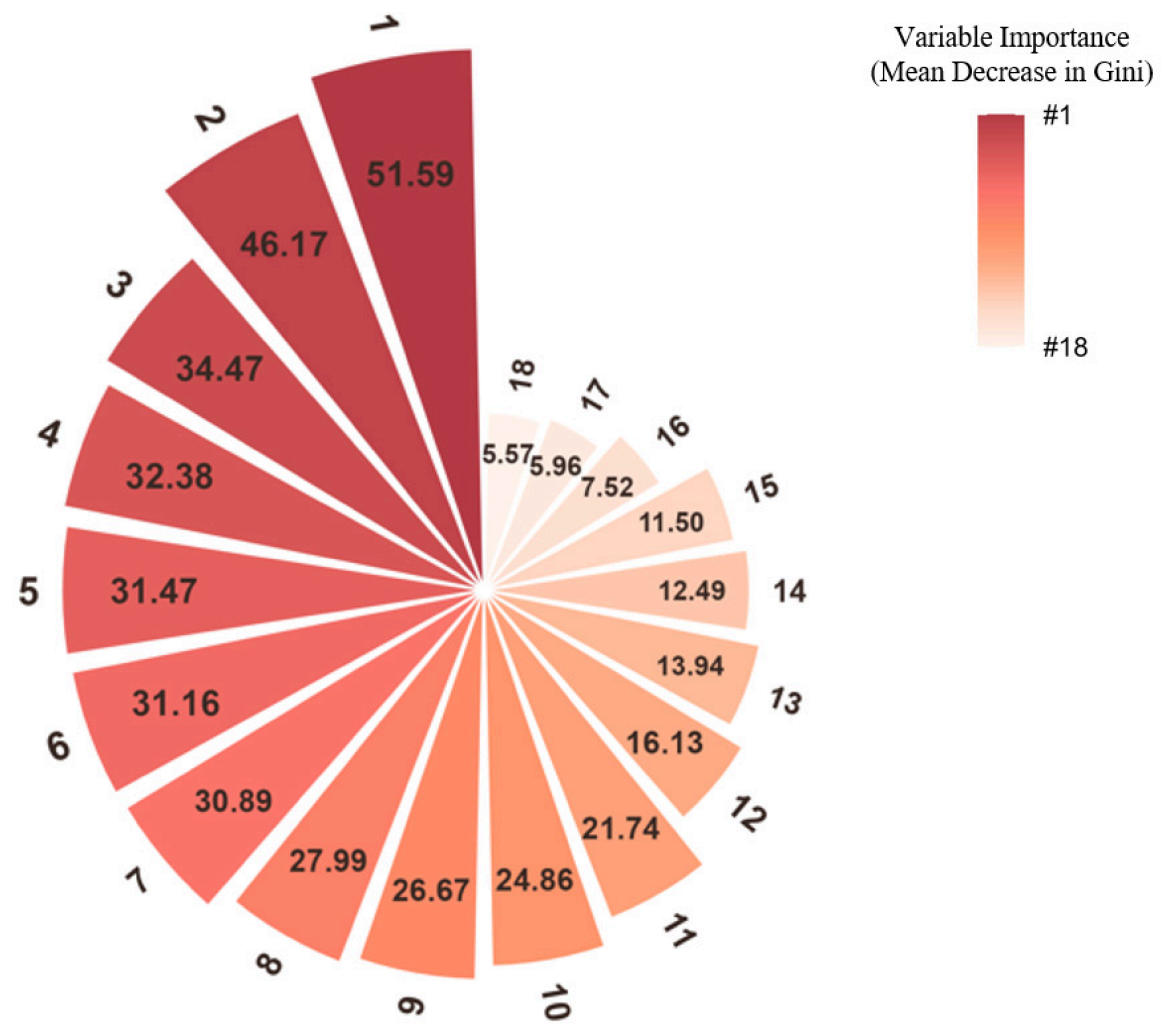
Random forest variable importance for the final 18-predictor model. Note. Numbers indicate the following predictors: 1, depressive symptoms; 2, 2.5-m walking test completion time; 3, left-hand grip strength; 4, self-rated health; 5, HDL cholesterol; 6, right-hand grip strength; 7, waist circumference; 8, age; 9, executive function; 10, sleep duration; 11, memory function; 12, distance vision; 13, near vision; 14, hearing status; 15, history of falls; 16, oral health status; 17, stroke; and 18, memory-related disease. # indicates the variable-importance rank.

In descending order of random forest importance, the final predictors were: 1, depressive symptoms; 2, 2.5-m walking test completion time; 3, left-hand grip strength; 4, self-rated health; 5, HDL cholesterol; 6, right-hand grip strength; 7, waist circumference; 8, age; 9, executive function; 10, sleep duration; 11, memory function; 12, distance vision; 13, near vision; 14, hearing status; 15, history of falls; 16, oral health status; 17, stroke; and 18, memory-related disease. Physical activity–related measures removed during the earlier missingness-screening stage were not re-introduced into the VIF or recursive feature-elimination procedures. The random forest model was subsequently refitted using the complete training set and these 18 predictors. Variable importance was quantified using the mean decrease in Gini impurity, with larger values indicating a greater contribution to node purity across the decision trees. As illustrated in Figure 4, depressive symptoms, 2.5-m walking test completion time, bilateral handgrip strength, self-rated health, HDL cholesterol, waist circumference, age, and executive function were among the most influential predictors. In the radial importance plot, bar radius was scaled according to the square root of the corresponding Gini-based importance value to reduce visual compression caused by highly influential predictors, whereas colour intensity represented the relative importance rank. All subsequent model development, model comparison, performance evaluation, and SHAP analyses were conducted using this optimized 18-predictor set.

### 3.3. Model Development and Initial Performance Evaluation

After feature selection, eight machine learning classification models were developed using the same 18 retained predictors: logistic regression, LASSO logistic regression, elastic net, random forest, XGBoost, generalized additive model (GAM), decision tree, and support vector machine (SVM). All models were trained under the original class distribution, without SMOTE, random over-sampling, random under-sampling, class weighting, or any other class-balancing procedure. Data preprocessing, standardization, and hyperparameter tuning were confined to the training set. The parameters estimated from the training set were then applied unchanged to the held-out internal test set. Models that depend on predictor scale (logistic regression, LASSO logistic regression, elastic net, GAM, and SVM) used training set-standardized predictors, whereas random forest, XGBoost, and decision tree used predictors on their original scales.

In the held-out internal test set (n = 247; 161 non-impaired and 86 impaired), random forest achieved the numerically highest AUC of 0.7735 (95% CI, 0.7139–0.8332), together with an accuracy of 0.7409, an F1 score of 0.5789, and the lowest Brier score of 0.1813. Logistic regression and GAM each yielded an AUC of 0.7583, followed by LASSO logistic regression (AUC = 0.7581), elastic net (AUC = 0.7580), SVM (AUC = 0.7554), XGBoost (AUC = 0.7358), and decision tree (AUC = 0.7062). The differences between random forest and the logistic-type and GAMs were modest, and their AUC confidence intervals overlapped substantially; therefore, the random forest result should be interpreted as a numerical advantage rather than evidence of marked superiority.

At the prespecified probability threshold of 0.50, sensitivity ranged from 0.3605 to 0.5116, whereas specificity ranged from 0.8075 to 0.8944 across the eight models. Random forest had a sensitivity of 0.5116 and a specificity of 0.8634. Thus, under the original class distribution, the models generally showed higher specificity than sensitivity, indicating that a substantial proportion of participants with concurrent functional impairment remained unidentified at the fixed threshold.

**Figure 5:**
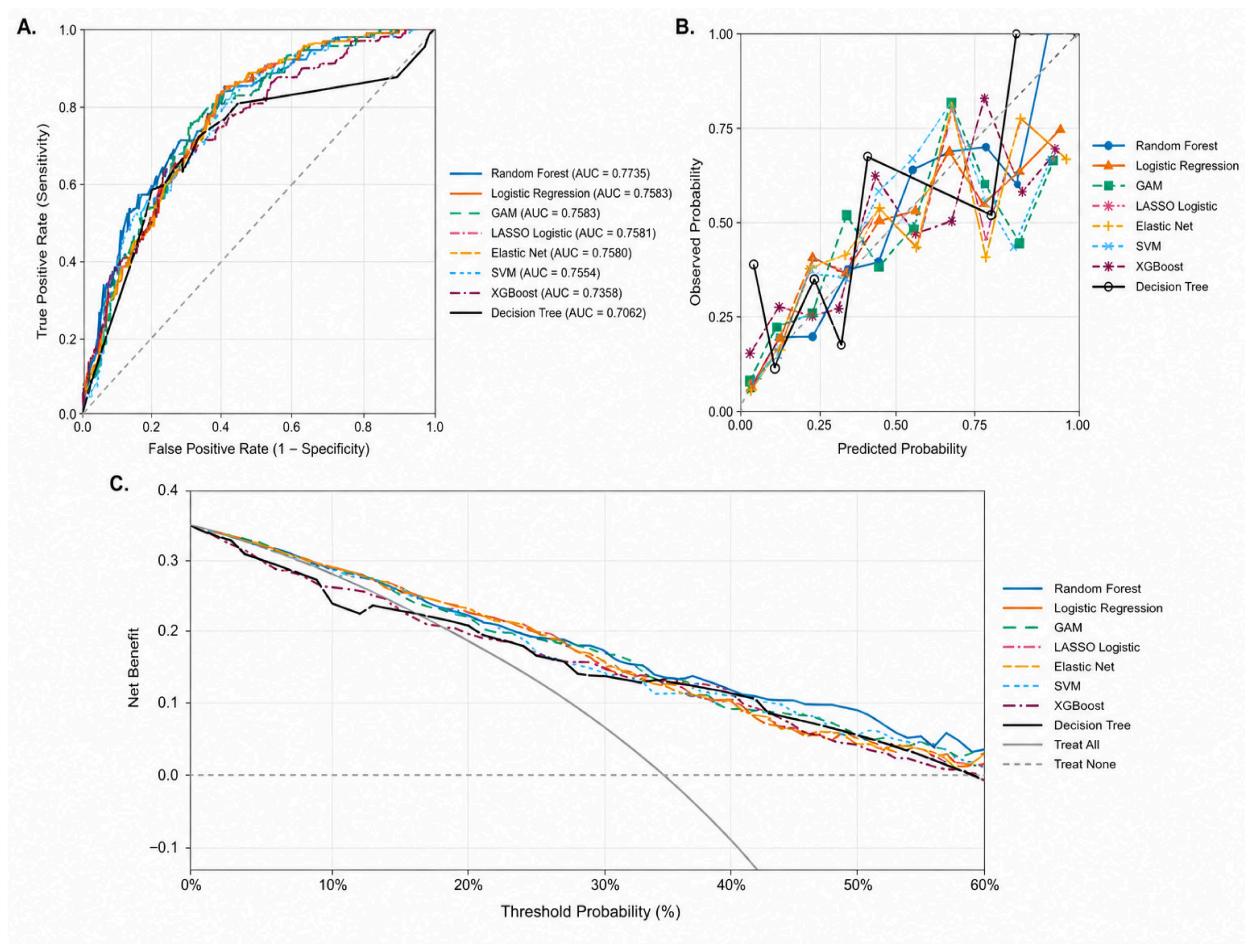
Performance of eight machine learning classification models for identifying concurrent functional impairment in the held-out internal test set. Note. (**A**) Receiver operating characteristic curves; (**B**) calibration curves; and (**C**) decision curves. The calibration points summarize 10 equal-width predicted-probability intervals, with point size proportional to the number of participants in each interval.

The ROC curves were consistent with these numerical findings (Figure 5A). The calibration plots showed increasing variability in the more sparsely populated high-probability bins (Figure 5B); among the eight models, random forest had the lowest Brier score (0.1813), whereas decision tree had the highest (0.2064). Decision curve analysis showed that all models provided positive net benefit and exceeded the “treat all” and “treat none” strategies over portions of the displayed threshold range (Figure 5C). At a threshold probability of 0.40, random forest and XGBoost each yielded a net benefit of 0.1174; at a threshold probability of 0.50, random forest yielded the highest net benefit of 0.0891. Considering its numerically highest AUC and accuracy, highest F1 score, lowest Brier score, and favorable decision-curve profile, random forest was selected for the subsequent interpretability analyses. This selection reflects its overall numerical performance in the internal held-out evaluation and does not establish superiority in an external population.

### 3.4. Repeated Resampling Stability Analysis Conditional on the Locked Feature Set

**Figure 6:**
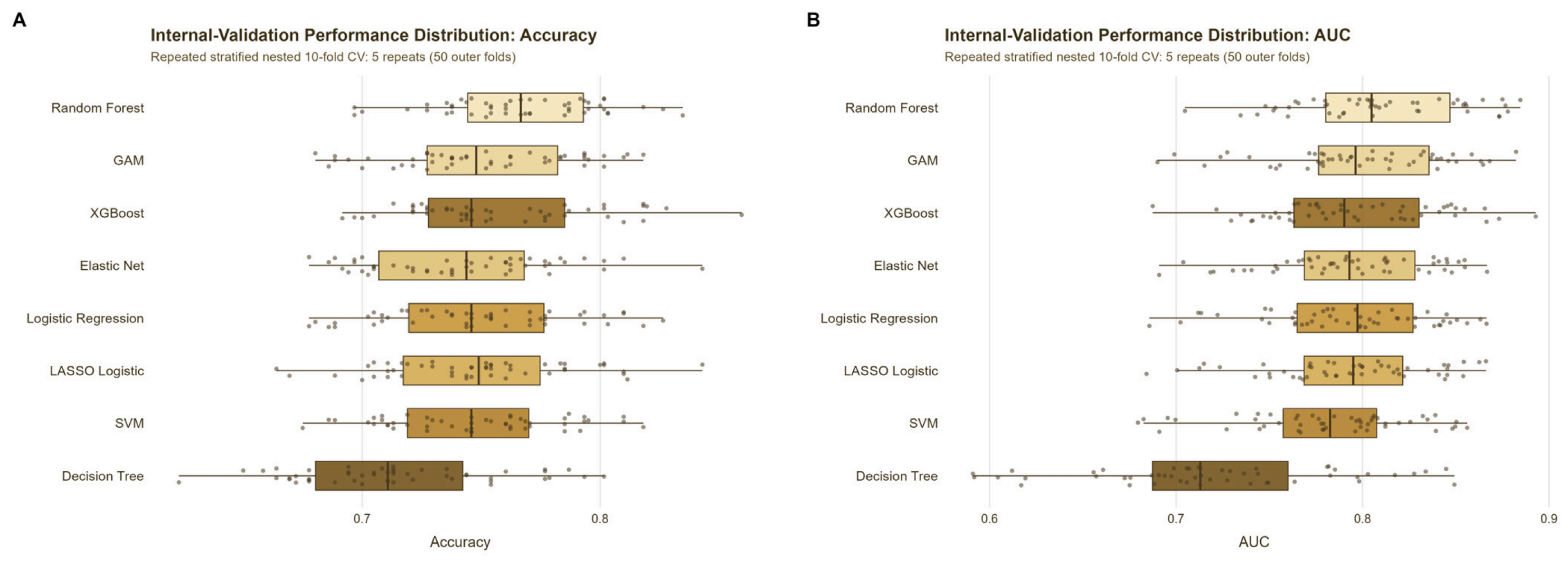
Internal-validation performance distributions across 50 outer test folds. Note. (**A**) Accuracy and (**B**) area under the receiver operating characteristic curve (AUC) for eight machine learning algorithms under five repeats of stratified nested 10-fold cross-validation.

To examine the stability of model performance under repeated resampling, the eight algorithms were evaluated in 50 outer test folds generated by five repeats of stratified nested 10-fold cross-validation. The repeated nested cross-validation analysis was conditional on the locked 18-predictor set and the previously completed imputed dataset. Within each outer fold, standardization parameters and model-specific hyperparameters were estimated using the outer training data only, whereas imputation and feature selection were not repeated. Model-specific results are presented in Table 2, and the distributions of accuracy and AUC across the 50 outer test folds are shown in Figure 6.

Random forest achieved the highest mean AUC (0.808 ± 0.043), the highest mean accuracy (0.766 ± 0.032), and the lowest Brier score (0.166 ± 0.016). Its mean sensitivity and specificity were 0.535 ± 0.072 and 0.887 ± 0.035, respectively. XGBoost produced a marginally higher mean F1 score than random forest (0.612 ± 0.065 vs. 0.609 ± 0.060) and higher sensitivity, but it had a lower mean AUC and a higher Brier score. GAM achieved a mean AUC of 0.799 ± 0.045; the three logistic-type models yielded similar mean AUCs of approximately 0.793. The decision tree showed the lowest mean AUC (0.720 ± 0.065). Overall, the resampling results supported random forest as the primary model on the basis of discrimination, accuracy, and probabilistic error, while also showing that its threshold-based sensitivity remained modest.

### 3.5. Sensitivity Analysis

In the prespecified sensitivity analysis, the random forest model was refitted after excluding 2.5-m walking test completion time and bilateral grip strength. Across the 50 outer folds, the reduced-input model retained most of the full model’s discrimination, although mean AUC decreased from 0.808 to 0.799 (mean difference, −0.009), accuracy decreased from 0.766 to 0.752, and the F1 score decreased from 0.609 to 0.584. The mean Brier score increased from 0.166 to 0.169. In the single held-out internal test set, the reduced-input model had an AUC of 0.781, accuracy of 0.729, sensitivity of 0.465, specificity of 0.870, F1 score of 0.544, and Brier score of 0.180. Although the AUC was numerically higher than that of the full model in the same test set (0.7735), this difference should not be interpreted as improved performance because it was based on a single split of 247 participants and may reflect sampling variability. These single-split results are reported separately from the repeated nested cross-validation estimates shown in Table 3. These findings suggest that most discrimination was retained without direct walking and grip-strength measurements, but threshold-dependent classification performance was modestly reduced under repeated resampling.

### 3.6. Interpretation of the Final Model Using SHAP

For interpretation only, the final random forest algorithm was refitted on the full analytic sample (*n* = 1213) using the locked 18-predictor set and the training-selected hyperparameters (mtry = 3; ntree = 500). No performance estimates were derived from this full-sample refit. The stratified SHAP background included 200 participants, and the resulting baseline model output was 0.34636. This value served only as the additive reference for SHAPs and was not interpreted as an external calibration estimate.

**Figure 7:**
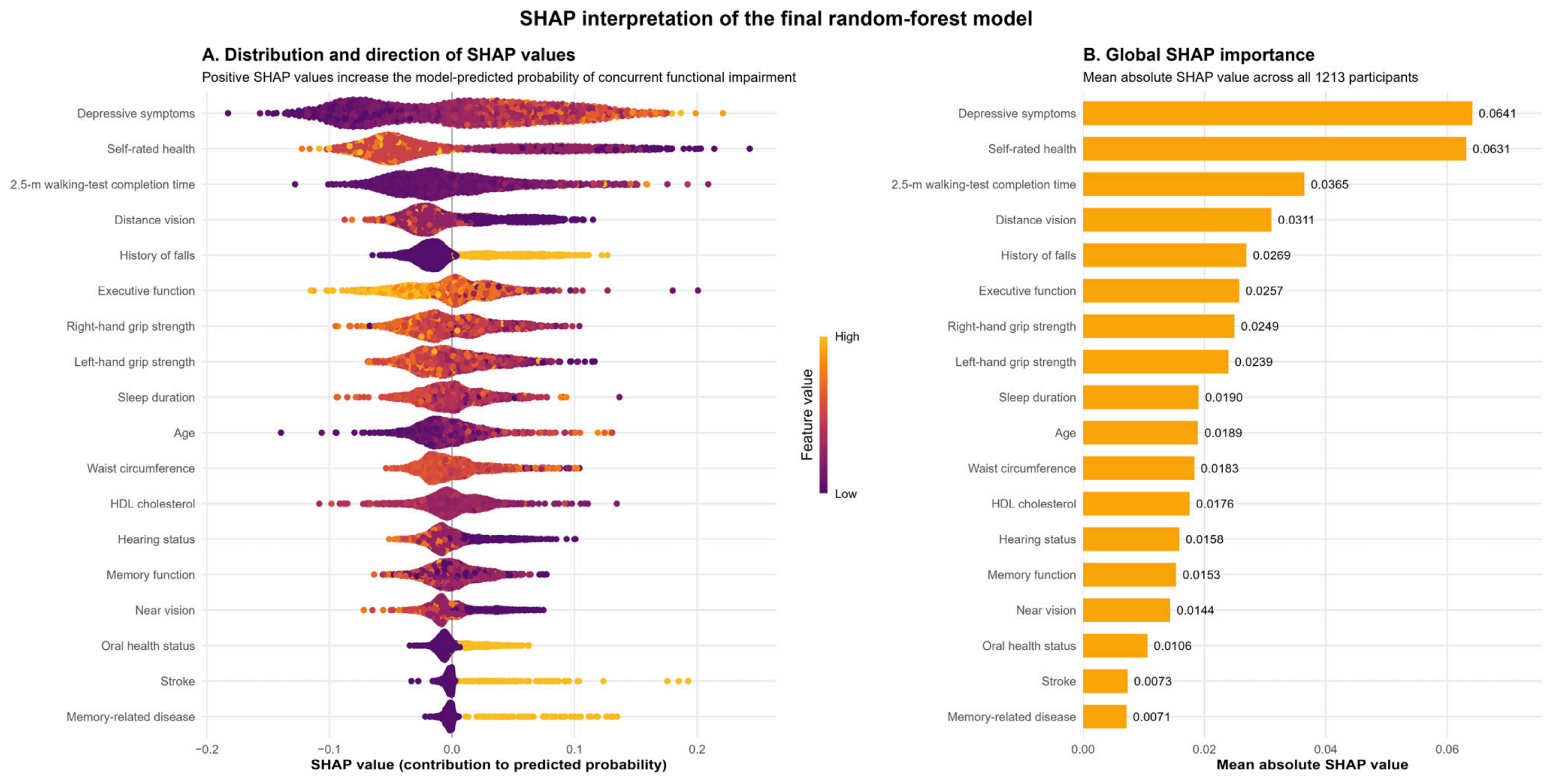
Global SHAP interpretation of the final random forest model. Note. (**A**) Beeswarm plot showing the distribution and direction of SHAP values for all 18 predictors. Each point represents one participant; color denotes the observed predictor value. Positive SHAP values increase the model-predicted probability of concurrent functional impairment, whereas negative values decrease it. (**B**) Global importance ranked by the mean absolute SHAP value across all 1213 participants.

The SHAP beeswarm plot showed substantial between-participant variation in both the magnitude and direction of feature contributions (Figure 7A). The eight highest global contributions, expressed as mean absolute SHAP values, were Depressive symptoms (0.0641), Self-rated health (0.0631), 2.5-m walking test completion time (0.0365), Distance vision (0.0311), History of falls (0.0269), Executive function (0.0257), Right-hand grip strength (0.0249), Left-hand grip strength (0.0239) (Figure 7B). Higher depressive-symptom scores and longer walking test completion times generally shifted model output toward the impaired class, whereas more favorable self-rated health, better distance vision and executive performance, and greater grip strength generally shifted model output toward the non-impaired class. These patterns describe the behavior of the fitted classifier and should not be interpreted as causal effects.

**Figure 8:**
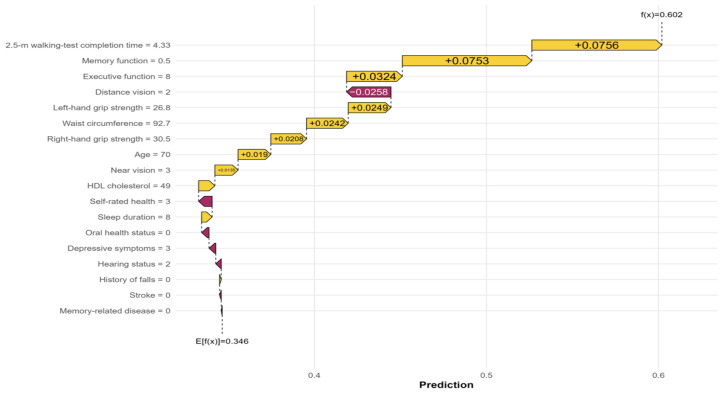
SHAP waterfall plot for the participant whose full-sample random forest output was closest to the prespecified 0.50 classification threshold. Note. *E*[*f*(*x*)] = 0.346 is the SHAP baseline model output, and *f*(*x*) = 0.602 is the final model-predicted probability of concurrent functional impairment for this participant. Positive contributions move the model output toward the impaired class, and negative contributions move it toward the non-impaired class. Yellow bars represent positive contributions that move the model output toward the impaired class, whereas dark-magenta bars represent negative contributions that move it toward the non-impaired class. Bar length indicates the magnitude of each contribution.

Local contributions also varied in composition across participants. The full-sample model output closest to the 0.50 classification threshold was 0.602 (Figure 8), relative to a SHAP baseline of 0.34636. For this participant, 2.5-m walking test completion time (SHAP = 0.07560), memory function (0.07531), executive function (0.03239), left-hand grip strength (0.02487), waist circumference (0.02421), right-hand grip strength (0.02077), and age (0.01904) shifted the model output upward, whereas distance vision (−0.02581), self-rated health (−0.00786), and several smaller contributions shifted it downward. The combined contributions produced the final model output of 0.602.

**Figure 9:**
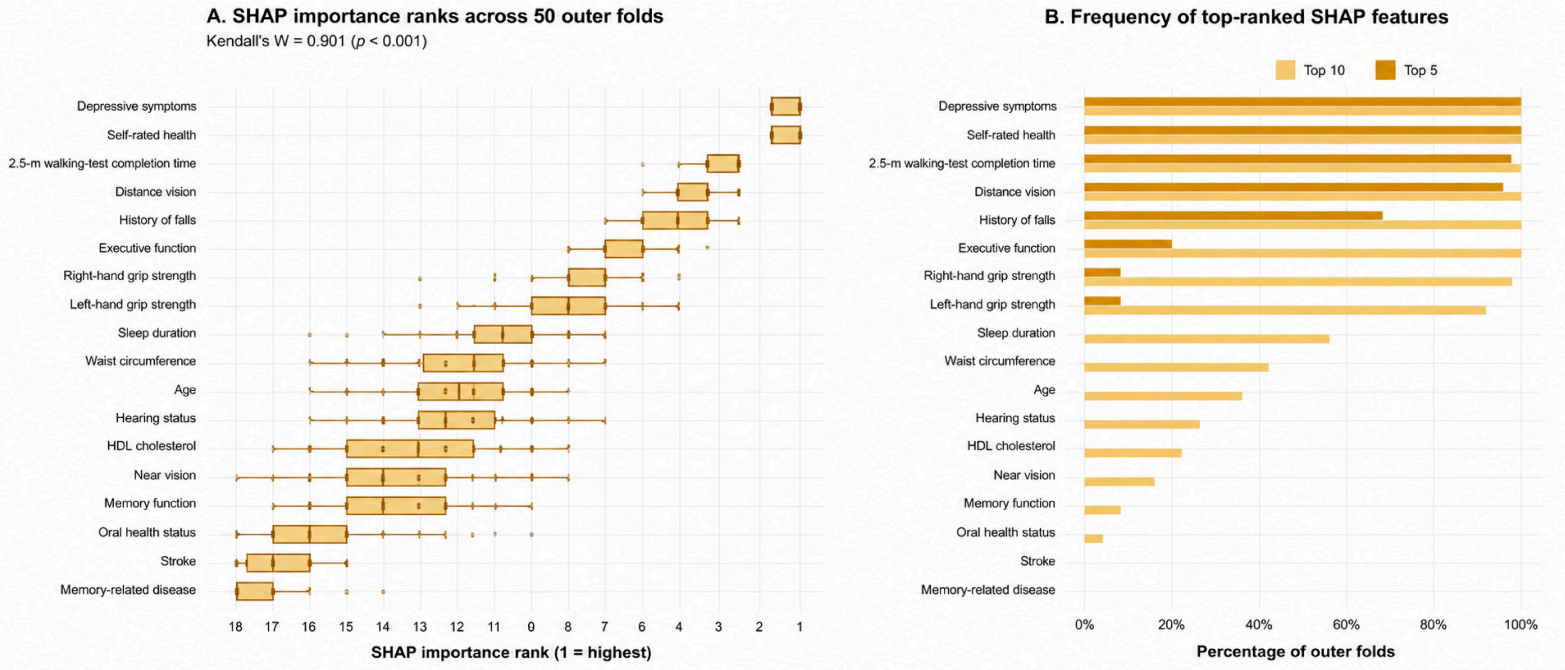
Stability of SHAP-based feature-importance rankings across the 50 outer test folds. Note. (**A**) Distribution of each predictor’s SHAP importance rank across five repeats of stratified 10-fold nested cross-validation. (**B**) Percentage of outer folds in which each predictor ranked among the top 5 or top 10.

SHAP importance rankings were highly consistent across the 50 outer test folds (Figure 9). Kendall’s W was 0.901, the median pairwise Spearman correlation between outer-fold rankings was 0.905, and the median Spearman correlation between each outer-fold ranking and the final full-sample ranking was 0.948. Depressive symptoms and self-rated health appeared in the top 5 in all 50 folds. Walking test completion time and distance vision appeared in the top 5 in 98% and 96% of folds, respectively, and history of falls did so in 70%. Depressive symptoms, self-rated health, walking test completion time, distance vision, history of falls, and executive function appeared in the top 10 in every fold; right- and left-hand grip strength appeared in the top 10 in 98% and 92% of folds, respectively.

## 4. Discussion

### 4.1. Main Findings

This study developed and internally evaluated machine learning models for identifying concurrent functional impairment among older adults with self-reported physician-diagnosed diabetes in CHARLS 2015. Among the algorithms examined, random forest achieved the most favorable overall balance across discrimination, calibration, probabilistic accuracy, and decision curve analysis, although its advantage over several competing models was modest. The final model integrated information from multiple domains, including psychological status, subjective health, physical performance, sensory function, cognition, and fall history. Depressive symptoms, self-rated health, 2.5-m walking test completion time, distance vision, history of falls, executive function, and bilateral grip strength were among the most influential features. Taken together, these findings suggest that functional impairment in older adults with diabetes is better characterized through a multidimensional profile than through any single clinical or functional indicator.

### 4.2. Psychological and Subjective-Health Features

Depressive symptoms and self-rated health made substantial contributions to model classification. Their prominence is clinically plausible because psychological well-being and perceived health are closely connected with everyday functioning. Depressive symptoms are often accompanied by reduced motivation, social withdrawal, lower participation in physical activity, and difficulty maintaining daily routines. At the same time, restrictions in activities of daily living may increase emotional distress, suggesting that the relationship between depressive symptoms and functional limitation is likely to be reciprocal [11,12,13].

Self-rated health may be informative because it summarizes several dimensions of health that are not fully captured by isolated clinical measurements. Individuals’ evaluations of their health can reflect physical symptoms, emotional burden, comorbid disease, social functioning, and recent changes in physical capacity [30]. Its contribution alongside objective performance and cognitive measures therefore suggests that subjective health perceptions contain complementary information about current functional vulnerability. Both depressive symptoms and self-rated health are also relatively easy to assess, making them practical indicators for identifying individuals who may require a more detailed functional evaluation.

### 4.3. Physical Performance and Fall-Related Features

Measures of physical performance were also central to the model. A longer 2.5-m walking test completion time represents slower walking performance, while lower grip strength indicates reduced upper-limb strength and may reflect broader declines in muscle function and physiological reserve. In older adults with diabetes, impaired mobility and muscle weakness may arise through several pathways, including reduced physical activity, neuropathy, vascular complications, inflammation, and age-related loss of muscle mass. Previous studies have similarly linked diabetes in later life with reduced strength, mobility limitation, falls, and disability [7,8,14,15].

The contribution of fall history is also clinically meaningful. Falls rarely reflect a single impairment; instead, they may summarize deficits in balance, lower-limb function, vision, cognition, medication tolerance, and environmental safety. For this reason, fall history may provide a concise indication of accumulated vulnerability across several physiological and behavioral systems.

Walking test performance, grip strength, and falls are particularly relevant to community and primary-care assessment because they are interpretable and comparatively simple to collect. However, these variables are also closely related to the ADL/IADL-based outcome and may partly reflect the same underlying functional construct. In the sensitivity analysis excluding direct physical performance measures, the model retained meaningful discrimination, although threshold-based classification performance generally declined. This finding suggests that a reduced-input model may remain useful when physical testing is not feasible, but the full model is preferable when these measures are available.

### 4.4. Cognitive, Sensory, and Physical Activity Findings

Executive function, memory-related measures, and sensory status extended the model beyond physical capability alone. Executive function supports planning, sequencing, attention, and adaptation during complex activities. Deficits in this domain may therefore interfere with medication management, financial tasks, travel, meal preparation, and other instrumental activities of daily living. The observed contribution of executive function is consistent with evidence linking diabetes to cognitive impairment and with the broader role of cognition in maintaining independence in later life [9,10].

Visual and hearing difficulties may also affect everyday functioning through several routes. Impaired vision can compromise navigation, reading, medication identification, and hazard detection, whereas hearing loss may interfere with communication, access to health information, and social participation. Sensory limitations may also increase the cognitive demands of routine tasks and contribute to reduced confidence in independent activity. The present findings are consistent with evidence that sensory impairment is associated with disability and shorter disability-free life expectancy [21,31].

Physical activity variables require more cautious interpretation because they were excluded before final feature ranking owing to extensive missingness, and the available measures were based on broad self-reported categories. Their absence from the final predictor set should therefore not be interpreted as evidence that physical activity is unrelated to functional health. Broad categories may not adequately represent differences in activity intensity, frequency, duration, or context, dimensions that are emphasized in physical activity guidance for older adults [32]. Nonlinear and threshold-dependent relationships may also be obscured by simple classification schemes. A study of older adults reported threshold and saturation patterns in the relationship between physical exercise and cognitive function [33]. Although that study examined a different population and outcome, it illustrates how coarse activity measures may fail to capture more complex activity–health relationships.

### 4.5. Value and Interpretation of the Machine Learning Framework

The machine learning framework allowed information from multiple health domains to be considered simultaneously and accommodated nonlinear associations and interactions that may be difficult to represent in conventional single-factor analyses. This is relevant in functional impairment, where physical performance, cognition, mood, sensory capacity, and chronic disease are likely to operate jointly rather than independently. Although random forest showed the most favorable internal performance in the present study, this finding should not be interpreted as evidence that machine learning methods are generally superior to logistic regression. A systematic review found no consistent performance benefit of machine learning over logistic regression across clinical prediction models [34].

The development and evaluation procedures were designed to reduce optimistic performance estimates. The training set was separated from the held-out internal test set, and preprocessing parameters were estimated using training data. Repeated stratified nested cross-validation provided an additional assessment of the stability of preprocessing and hyperparameter tuning. Feature selection, however, was not repeated within each outer fold. The held-out test-set results should therefore be regarded as the primary post-selection performance estimates, while the nested cross-validation results provide supplementary evidence regarding model stability [22,23,24].

Model evaluation included discrimination, calibration, Brier score, threshold-based classification measures, and decision curve analysis. Considering these metrics together is important because good discrimination alone does not ensure reliable probability estimates or meaningful net benefit across clinically relevant thresholds [27,28,29]. The original class distribution was retained without SMOTE or other balancing procedures, thereby preserving the probability scale used in the primary analysis.

SHAP analysis was used to clarify how individual features contributed to the fitted random forest output, and the stability analysis assessed whether the broad ranking of influential variables was dependent on a single data split [19]. These results improve model interpretability, but SHAP values describe contributions to model output rather than causal effects. Their direction and magnitude must also be interpreted according to the original coding and scale of each variable.

### 4.6. Clinical and Public Health Implications

The final model may help identify older adults with diabetes who have a higher probability of current functional impairment and who may benefit from formal assessment. Several of the most influential inputs, including depressive symptoms, self-rated health, walking test completion time, grip strength, cognitive performance, and sensory status, can be collected in community or primary-care settings with limited equipment. A multidomain approach may therefore be more informative than screening based solely on age, comorbidity, or laboratory indicators.

At its current stage, the model should be viewed as a research-based screening aid rather than a diagnostic instrument. The observed sensitivity does not support stand-alone case finding, and the model was evaluated only within the CHARLS source population. Before practical implementation, its performance should be examined in independent cohorts, and decision thresholds should be selected according to the consequences of false-positive and false-negative classifications [35,36,37].

## 5. Limitations

Several limitations should be considered. First, the cross-sectional design does not establish temporal sequence. The model classifies functional impairment present at the time of the 2015 assessment and does not estimate the future incidence of functional decline. Relationships between functional status and features such as depressive symptoms, self-rated health, cognition, and physical performance may also operate in both directions.

Second, diabetes status, disease history, physical activity, self-rated health, and several other variables were based partly on self-report and may be affected by recall or reporting bias. CHARLS may not include all clinical, environmental, treatment-related, behavioral, and social factors relevant to functional impairment, and some available variables were not retained in the final model.

Third, exclusions due to unavailable outcome data and extensive predictor missingness may have introduced selection bias. Training-based imputation reduced the risk of information leakage but could not recover unobserved information or address bias arising from data that were not missing at random. Extreme observations, measurement error, and residual uncertainty in variable coding may also have influenced feature selection and model output despite the data-quality checks undertaken.

Fourth, although the source cohort used a national sampling framework, the present analysis was based on an unweighted subgroup of eligible Chinese older adults with self-reported physician-diagnosed diabetes. The results should therefore not be interpreted as nationally representative. Generalizability may be limited for the frailest individuals, younger populations, people from other countries, and patients with clinically ascertained diabetes.

Finally, all model development and evaluation were conducted using the same survey wave and source population. The held-out test set and repeated nested cross-validation provide internal rather than external validation, and the resampling analysis was conditional on the previously selected predictor set. Performance across demographic or clinical subgroups was not comprehensively assessed. Independent external cohorts and prospective studies are needed to examine transportability, calibration, subgroup performance, and real-world usefulness.

## 6. Conclusions

Random forest showed the most favorable overall internal performance for classifying concurrent functional impairment among older adults with self-reported diabetes. Psychological status, subjective health, physical performance, sensory function, cognition, and fall history all contributed to classification, underscoring the multidimensional nature of functional vulnerability in this population. These findings support the use of integrated functional assessment in community and primary-care settings. External validation and prospective evaluation are required before the model can be considered for clinical implementation.

## Figures and Tables

**Table 1:** Baseline characteristics of the participants (*n* = 1213).

Variable	Overall (*n* = 1213)	No Impairment (*n* = 798)	Impairment (*n* = 415)	*p*-Value
1 Age, years	71.89 ± 5.84	71.32 ± 5.46	72.98 ± 6.36	<0.001
2 Sex (%)				0.020
Female	678 (55.89)	427 (53.51)	251 (60.48)	
Male	535 (44.11)	371 (46.49)	164 (39.52)	
3 Any chronic disease (%)				0.003
No	48 (4.09)	41 (5.36)	7 (1.72)	
Yes	1125 (95.91)	724 (94.64)	401 (98.28)	
4 Hypertension (%)				0.098
No	444 (38.98)	303 (40.73)	141 (35.70)	
Yes	695 (61.02)	441 (59.27)	254 (64.30)	
5 Heart disease (%)				0.001
No	752 (66.14)	514 (69.46)	238 (59.95)	
Yes	385 (33.86)	226 (30.54)	159 (40.05)	
6 Stroke (%)				<0.001
No	1023 (89.34)	694 (92.41)	329 (83.50)	
Yes	122 (10.66)	57 (7.59)	65 (16.50)	
7 Arthritis or rheumatism (%)				<0.001
No	553 (48.51)	398 (53.49)	155 (39.14)	
Yes	587 (51.49)	346 (46.51)	241 (60.86)	
8 Kidney disease (%)				<0.001
No	968 (85.06)	653 (87.65)	315 (80.15)	
Yes	170 (14.94)	92 (12.35)	78 (19.85)	
9 Psychiatric or emotional problems (%)				0.001
No	1108 (96.94)	734 (98.13)	374 (94.68)	
Yes	35 (3.06)	14 (1.87)	21 (5.32)	
10 Memory-related disease (%)				<0.001
No	1058 (92.97)	720 (96.13)	338 (86.89)	
Yes	80 (7.03)	29 (3.87)	51 (13.11)	
11 Dyslipidemia (%)				0.590
No	705 (63.29)	468 (63.85)	237 (62.20)	
Yes	409 (36.71)	265 (36.15)	144 (37.80)	
12 Lung disease (%)				0.004
No	922 (80.24)	622 (82.71)	300 (75.57)	
Yes	227 (19.76)	130 (17.29)	97 (24.43)	
13 Vigorous physical activity (%)				0.782
No	431 (79.52)	297 (79.20)	134 (80.24)	
Yes	111 (20.48)	78 (20.80)	33 (19.76)	
14 Moderate physical activity (%)				<0.001
No	309 (57.12)	196 (52.41)	113 (67.66)	
Yes	232 (42.88)	178 (47.59)	54 (32.34)	
15 Light physical activity (%)				<0.001
No	128 (23.66)	60 (16.00)	68 (40.96)	
Yes	413 (76.34)	315 (84.00)	98 (59.04)	
16 Regular exercise (%)				<0.001
No	95 (17.56)	40 (10.67)	55 (33.13)	
Yes	446 (82.44)	335 (89.33)	111 (66.87)	
17 Lost all teeth/severe oral problem (%)				<0.001
No	880 (72.55)	614 (76.94)	266 (64.10)	
Yes	333 (27.45)	184 (23.06)	149 (35.90)	
18 History of falls (%)				<0.001
No	893 (73.68)	642 (80.45)	251 (60.63)	
Yes	319 (26.32)	156 (19.55)	163 (39.37)	
19 Alcohol drinking (%)				0.006
No	888 (73.21)	564 (70.68)	324 (78.07)	
Yes	325 (26.79)	234 (29.32)	91 (21.93)	
20 Smoking (%)				0.087
No	941 (77.64)	607 (76.16)	334 (80.48)	
Yes	271 (22.36)	190 (23.84)	81 (19.52)	
21 Cancer or malignant tumor (%)				0.233
No	1122 (97.91)	739 (98.27)	383 (97.21)	
Yes	24 (2.09)	13 (1.73)	11 (2.79)	
22 Liver disease (%)				0.355
No	1048 (92.09)	691 (92.63)	357 (91.07)	
Yes	90 (7.91)	55 (7.37)	35 (8.93)	
23 Digestive disease (%)				0.001
No	759 (66.29)	522 (69.60)	237 (60.00)	
Yes	386 (33.71)	228 (30.40)	158 (40.00)	
24 Asthma (%)				<0.001
No	1019 (89.00)	686 (91.71)	333 (83.88)	
Yes	126 (11.00)	62 (8.29)	64 (16.12)	
25 Self-rated health (%)				<0.001
Very poor	98 (8.61)	30 (3.88)	68 (18.68)	
Poor	299 (26.27)	146 (18.86)	153 (42.03)	
Fair	571 (50.18)	457 (59.04)	114 (31.32)	
Good	105 (9.23)	85 (10.98)	20 (5.49)	
Very good	65 (5.71)	56 (7.24)	9 (2.47)	
26 Distance vision (%)				<0.001
Poor	375 (32.92)	180 (23.26)	195 (53.42)	
Fair	528 (46.36)	405 (52.33)	123 (33.70)	
Good	115 (10.10)	88 (11.37)	27 (7.40)	
Very good	102 (8.96)	86 (11.11)	16 (4.38)	
Excellent	19 (1.67)	15 (1.94)	4 (1.10)	
27 Near vision (%)				<0.001
Poor	268 (23.57)	129 (16.69)	139 (38.19)	
Fair	594 (52.24)	431 (55.76)	163 (44.78)	
Good	155 (13.63)	119 (15.39)	36 (9.89)	
Very good	101 (8.88)	80 (10.35)	21 (5.77)	
Excellent	19 (1.67)	14 (1.81)	5 (1.37)	
28 Hearing (%)				<0.001
Poor	252 (22.11)	126 (16.26)	126 (34.52)	
Fair	617 (54.12)	439 (56.65)	178 (48.77)	
Good	144 (12.63)	109 (14.06)	35 (9.59)	
Very good	117 (10.26)	93 (12.00)	24 (6.58)	
Excellent	10 (0.88)	8 (1.03)	2 (0.55)	
29 Total physical activity (MET-related summary)	4511.40 ± 5445.29 (*n* = 522)	4858.90 ± 5401.59 (*n* = 361)	3732.22 ± 5479.00 (*n* = 161)	0.030
30 Sleep duration, hours	6.14 ± 2.20	6.34 ± 2.05	5.69 ± 2.43	<0.001
31 Depressive symptoms score	9.16 ± 6.54	7.58 ± 5.90	12.54 ± 6.58	<0.001
32 Memory score	2.55 ± 1.78	2.74 ± 1.83	2.15 ± 1.61	<0.001
33 Executive function score	7.78 ± 2.66	8.17 ± 2.50	6.81 ± 2.80	<0.001
34 Global cognitive function score	10.65 ± 3.74	11.15 ± 3.64	9.34 ± 3.68	<0.001
35 2.5-m walking test completion time, seconds	3.96 ± 2.04	3.58 ± 1.31	4.82 ± 2.91	<0.001
36 Left-hand grip strength, kg	22.45 ± 9.48	23.88 ± 9.25	19.47 ± 9.25	<0.001
37 Right-hand grip strength, kg	24.04 ± 9.81	25.41 ± 9.74	21.17 ± 9.34	<0.001
38 Peak expiratory flow	234.64 ± 108.07	245.61 ± 108.84	211.69 ± 102.87	<0.001
39 Systolic blood pressure, mmHg	137.88 ± 21.36	137.81 ± 20.41	138.02 ± 23.17	0.882
40 Diastolic blood pressure, mmHg	74.70 ± 10.96	74.54 ± 10.70	75.01 ± 11.47	0.528
41 BMI, kg/m^2^	24.52 ± 3.94	24.61 ± 3.77	24.33 ± 4.27	0.302
42 Waist circumference, cm	88.52 ± 15.43	89.09 ± 14.27	87.34 ± 17.56	0.110
43 Glucose, mg/dL	136.30 ± 60.66	135.98 ± 59.76	136.98 ± 62.65	0.813
44 Glycated hemoglobin, %	7.15 ± 1.53	7.12 ± 1.44	7.23 ± 1.71	0.324
45 Blood urea nitrogen, mg/dL	16.34 ± 5.02	16.16 ± 4.63	16.71 ± 5.76	0.143
46 Creatinine, mg/dL	0.84 ± 0.30	0.85 ± 0.30	0.83 ± 0.31	0.469
47 Cystatin C, mg/L	1.00 ± 0.31	0.98 ± 0.26	1.04 ± 0.40	0.011
48 C-reactive protein, mg/L	4.01 ± 9.32	3.54 ± 7.67	5.05 ± 12.08	0.043
49 Triglyceride-glucose index	9.09 ± 0.69	9.09 ± 0.68	9.09 ± 0.69	0.921
50 TyG-BMI	224.44 ± 43.59	224.65 ± 42.09	223.94 ± 46.91	0.825
51 Triglycerides, mg/dL	159.73 ± 92.41	159.15 ± 92.05	160.99 ± 93.31	0.774
52 HDL cholesterol, mg/dL	49.17 ± 11.88	49.36 ± 11.44	48.76 ± 12.80	0.482
53 LDL cholesterol, mg/dL	106.44 ± 30.23	106.25 ± 29.29	106.85 ± 32.19	0.779
54 Hemoglobin, g/dL	13.59 ± 1.91	13.66 ± 1.84	13.44 ± 2.06	0.118

Note: Values are presented as mean ± SD or *n* (%). *p*-values were obtained using Welch’s *t*-test, chi-square test, or Fisher’s exact test, as appropriate.

**Table 2:** Repeated resampling stability of different machine learning models, conditional on the locked feature set, across 50 outer test folds.

Model	AUC	Accuracy	Sensitivity	Specificity	Precision	F1 Score	Brier Score
Random Forest	0.808 ± 0.043	0.766 ± 0.032	0.535 ± 0.072	0.887 ± 0.035	0.714 ± 0.066	0.609 ± 0.060	0.166 ± 0.016
GAM	0.799 ± 0.045	0.751 ± 0.037	0.523 ± 0.086	0.869 ± 0.039	0.678 ± 0.071	0.587 ± 0.070	0.169 ± 0.018
XGBoost	0.796 ± 0.045	0.758 ± 0.039	0.558 ± 0.077	0.863 ± 0.043	0.683 ± 0.077	0.612 ± 0.065	0.170 ± 0.021
Elastic Net	0.793 ± 0.043	0.743 ± 0.038	0.424 ± 0.092	0.909 ± 0.039	0.713 ± 0.096	0.526 ± 0.084	0.174 ± 0.014
Logistic Regression	0.793 ± 0.043	0.748 ± 0.038	0.505 ± 0.082	0.875 ± 0.041	0.681 ± 0.079	0.576 ± 0.070	0.171 ± 0.018
LASSO Logistic	0.793 ± 0.043	0.748 ± 0.038	0.483 ± 0.083	0.886 ± 0.036	0.690 ± 0.081	0.566 ± 0.074	0.171 ± 0.016
SVM	0.782 ± 0.045	0.748 ± 0.035	0.492 ± 0.076	0.881 ± 0.039	0.687 ± 0.075	0.570 ± 0.066	0.175 ± 0.017
Decision Tree	0.720 ± 0.065	0.714 ± 0.041	0.506 ± 0.112	0.823 ± 0.057	0.601 ± 0.073	0.543 ± 0.083	0.199 ± 0.023

Note. Values are mean ± SD across five repeats of stratified nested 10-fold cross-validation (50 outer test folds).

**Table 3:** Sensitivity analysis of the full and reduced random forest models under repeated nested 10-fold cross-validation.

Metric	Full RF, Mean	Reduced RF, Mean	Reduced − Full
AUC	0.808	0.799	−0.009
Accuracy	0.766	0.752	−0.014
Sensitivity	0.535	0.513	−0.022
Specificity	0.887	0.876	−0.011
F1 score	0.609	0.584	−0.025
Brier score	0.166	0.169	0.003

Note: Values are means across five repeats of stratified nested 10-fold cross-validation, yielding 50 outer test folds.

## Data Availability

CHARLS datasets are available for download at the CHARLS home website (http://charls.pku.edu.cn/en, accessed on 15 April 2026).
